# Diminishing returns: A comparison between fresh mass vs. area and dry mass vs. area in deciduous species

**DOI:** 10.3389/fpls.2022.832300

**Published:** 2022-10-04

**Authors:** Xuchen Guo, Karl J. Niklas, Yirong Li, Jianhui Xue, Peijian Shi, Julian Schrader

**Affiliations:** ^1^ Bamboo Research Institution, College of Biology and the Environment, Nanjing Forestry University, Nanjing, China; ^2^ School of Integrative Plant Science, Cornell University, Ithaca, NY, United States; ^3^ College of Life Science, South China Agricultural University, Guangzhou, China; ^4^ Institute of Botany, Jiangsu Province and Chinese Academy Sciences, Nanjing, China; ^5^ School of Natural Sciences, Macquarie University, Sydney, NSW, Australia; ^6^ Biodiversity, Macroecology and Biogeography, University of Göttingen, Göttingen, Germany

**Keywords:** diminishing returns, leaf area, leaf biomass, light-capture, water content

## Abstract

“Diminishing returns” in leaf economics occurs when increases in lamina mass (*M*), which can either be represented by lamina dry mass (DM) or fresh mass (FM), fail to produce proportional increases in leaf surface area (*A*), such that the scaling exponent (α) for the *M* vs. *A* scaling relationship exceeds unity (i.e., α > 1.0). Prior studies have shown that FM vs. *A* is better than DM vs *A* in assessing diminishing returns in evergreen species. However, the superiority of FM vs. *A* over DM vs. *A* has been less well examined for deciduous species. Here, we applied reduced major axis protocols to test whether FM vs. *A* is better than DM vs. *A* to describe the *M* vs. *A* scaling relationship, using a total of 4271 leaves from ten deciduous and two evergreen tree species in the Fagaceae and Ulmaceae for comparison. The significance of the difference between the scaling exponents of FM vs. *A* and DM vs. *A* was tested using the bootstrap percentile method. Further, we tested the non-linearity of the FM (DM) vs. *A* data on a log-log scale using ordinary least squares. We found that (i) the majority of scaling exponents of FM vs. *A* and DM vs. *A* were >1 thereby confirming diminishing returns for all 12 species, (ii) FM vs. *A* was more robust than DM vs. *A* to identify the *M* vs. *A* scaling relationship, (iii) the non-linearity of the allometric model was significant for both DM vs. *A* and FM vs. *A*., and (iv) the evergreen species of Fagaceae had significantly higher DM and FM per unit area than other deciduous species. In summary, FM vs. *A* is a more reliable measure than DM vs. *A* when dealing with diminishing returns, and deciduous species tend to invest less biomass in unit leaf light harvesting area than evergreen species.

## Introduction

Leaves are the primary light-harvesting organs of most vascular land plants. They convert solar irradiance into chemical energy by means of photosynthesis (Rascher and Nedbal, 2006). As such, the biology of leaves provides deep insights into plant economic spectra and ecological strategies (Westoby et al., 2002).

Leaf functional traits (leaf area, leaf mass and leaf water content, etc.) are related to plant growth strategies as well as to ecosystem processes such as primary productivity and nutrient cycling (Garnier et al., 2001; Wright et al., 2004). Scaling relationships of leaf functional traits, such as leaf dimensions and leaf mass, are useful to understand the full spectrum of leaf forms and functions as well as to characterize evolutionary stable leaf forms (Niklas et al., 2007; Shi et al., 2019).

For example, leaf mass (*M*) and leaf lamina surface area (*A*) follow a quantifiable scaling relationship described by the power-law function *M* = β*A^a^*, where β is the normalization constant, and α is the scaling exponent. For many plant species groups, the numerical values of α exceed unity, which indicates that leaf mass increases at a faster rate than leaf area, a phenomenon referred to as “diminishing returns” (Milla and Reich, 2007; Niklas et al., 2007; Sun et al., 2017). A widely accepted explanation is that larger leaves require a disproportionately larger amount of non-photosynthetic tissues, which results in an increased biomass-cost to support leaf area (Niklas, 1994; Niklas et al., 2009; Sack et al., 2012; Shi et al., 2022a). The concept of diminishing returns is important to understand the energy allocation and variation among leaves from different species, which can affect ecosystem processes especially light capture (Westoby and Wright, 2003; Koester et al., 2014). In addition, the phenomenon of diminishing returns between *M* and *A* reflects a series of trade-offs, such as biomass allocation to the lamina with respect to biomass allocation to the petiole and lamina midrib (Niklas, 1991; Niklas, 1992; Niinemets et al., 2006).

Previous studies have used leaf dry mass (DM) as a measure of leaf biomass. However, the numerical value of the exponent governing the DM vs. *A* scaling relationship is not independent of the environment (Pan et al., 2013; Sun et al., 2017), and the water content of the lamina is an additional mechanical load that must be supported by the petiole (Niklas, 1991; Niklas, 1992; Li et al., 2022). Plants must optimize energy allocation among different structures and often allocate more biomass to the compartments responsive to environmental changes (Enquist et al., 2007; Price and Enquist, 2007; Zhang et al., 2011). For example, in some extreme circumstances, such as a windy environment, leaves may increase the proportion of biomass allocation to the lamina support tissues to provide sufficient mechanical stability; or under drought, biomass allocation may be devoted to root growth (Niklas and Enquist, 2002). Consequently, the extent to which leaves manifest diminishing returns is related to the environmental characteristics of a habitat (Takenaka et al., 2001).

In order to study diminishing returns under different habitats, investigators have examined the effects of altitude on the scaling of DM vs. *A*, because environmental factors, such as temperature or precipitation, can rapidly change over short distances along an elevational gradient (Pan et al., 2013). For example, Li et al. (2008) found that the scaling exponent of DM vs. *A* always exceeds unity for 93 temperate woody species collected at different elevations. However, Pan et al. (2013) analyzed the scaling relationships between DM and *A* for 121 vascular plant species along an elevational gradient in a subtropical monsoon forest, and found that the numerical values of the scaling exponent increased significantly with altitude from a numerical value of 0.859 to 1.299, with exponents consistently<1 in low altitudes. The disagreement between these two studies can be quantified by the extent of the goodness of fit for the different datasets. In the case of Pan et al. (2013), the value *r* ^2^<0.8 is significantly smaller than that of *r* ^2^ >0.9 reported by Li et al. (2008), which suggests that DM vs. *A* might be unreliable for describing the scaling of *M* vs. *A* under some circumstances. Thus, investigators have sought to find a substitute for DM to evaluate *M* vs. *A*.

Considering that the water in leaves is metabolically essential and that it contributes to the mechanical loads that the petiole (and the secondary and midrib veins) must structurally support, some studies have proposed that FM might be better than DM to assess *M* vs. *A* scaling relationships. For example, using FM vs. *A* as opposed to DM vs. *A* is statistically more robust when assessing the *M* vs. *A* scaling relationship for bamboo leaves (Shi et al., 2015; Lin et al., 2018). Likewise, (Huang et al., 2019a; Huang et al., 2019b) compared the scaling relationship between FM vs. *A* with that of DM vs. *A* using 15 broad-leaved species and 12 bamboo species, which found the same phenomenon.

Prior studies have compiled large datasets on the size of individual evergreen leaves tree species, log-transformed both variables of interest, have applied linear regression of the pooled data to compare the difference between FM vs. *A* and DM vs. *A*, and have concluded that FM vs. *A* is more reliable when dealing with “diminishing returns” because FM vs. *A* has a better goodness of fit compared to DM vs. *A* (see Huang et al., 2019a; Huang et al., 2019b). However, this approach assumes that the relationship is log-log linear. If the relationship has other forms (e.g., quadratic rather than linear), this assumption has consequences for the estimated slope and the goodness of fit, which may be unpersuasive or inconclusive.

Yet another concern about the assertion that FM vs. *A* is more reliable when dealing with “diminishing returns” is that most studies have focused on evergreen species. Evergreen species retain their leaves for several years, and prior work has shown a huge range of leaf life spans among evergreen woody species, ranging from a couple of months in some tropical pioneer species to > 20 years in some gymnosperms (Chabot and Hicks, 1982; Reich et al., 1992). Some evergreen species are “leaf-exchangers”, dropping most of the previous season’s leaves just as the new cohort emerges (Lusk, 2019). Compared to evergreen species, deciduous species shed their leaves at the end of the growing season (Zhang et al., 2017). Thus, deciduous species may not invest as much biomass in leaf area expansion as evergreen species because their leaves are more ‘disposable’ (Athokpam et al., 2014). Alternatively, deciduous species may invest more biomass in leaf area expansion than evergreen species as their relatively short leaf lifespan requires light harvesting and more energy storage in relatively short time spans (Poorter et al., 2009; Tomlinson et al., 2013). Arguably, the scaling relationship for “diminishing returns” may be different in deciduous and evergreen species.

Here, we examine ten deciduous and two evergreen species to compare the difference between FM vs. *A* and DM vs. *A*, and test the non-linearity of traditional linear regression models. The species examined are from two families, the Fagaceae and Ulmaceae, that contain keystone species in forest ecosystems (Kremer et al., 2012; Fragnière et al., 2021). The taxonomic focus on these two families also permits a comparison of deciduous and evergreen species within a single family (i.e., the Fabaceae), thereby removing the effects of phylogenetic bias.

In these comparisons, we asked two questions: (1) is FM vs. *A* more reliable than DM vs. *A* for the description of diminishing returns in deciduous species?, and (2) does leaf biomass investment strategy differ between deciduous and evergreen species?

## Materials and methods

### Collection site and plant materials

**Figure 1:**
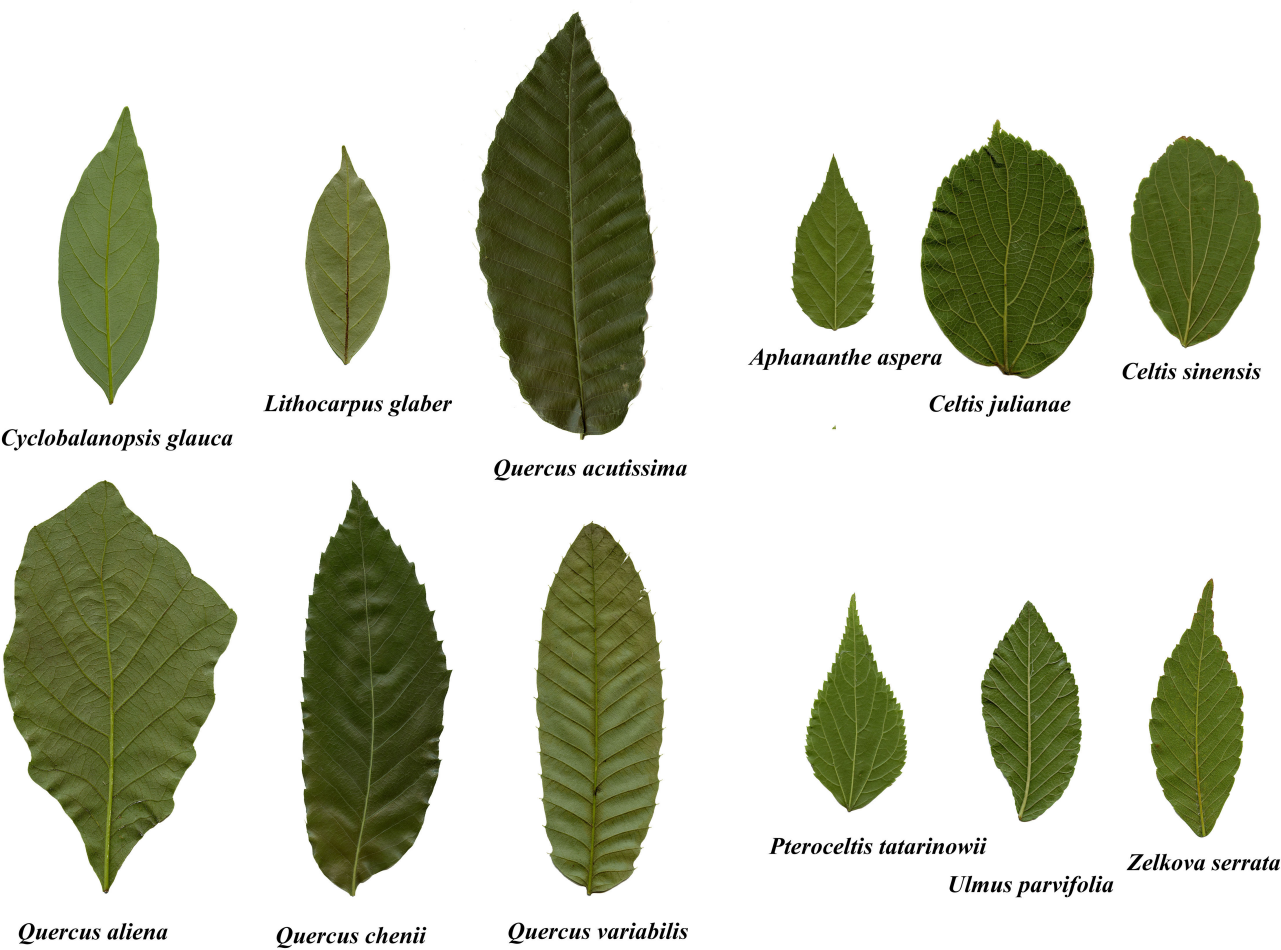
Examples of the leaves of the 12 species investigated in this study.

A total of 4271 mature and undamaged leaves was collected from Nanjing Forestry University (32°07’67”N, 118°81’36”E), Nanjing, Jiangsu Province, China. Given the possible influences of seasons on the scaling exponent of leaf mass vs. *A* for deciduous trees (Liu et al., 2020), leaves were collected in a short time period, from 20 August to 3 September 2020. For each species, >300 leaves were collected in the morning (9:30-11:30 am) from five to ten free standing trees. To reduce water loss during transport, leaves were wrapped in wet paper and then placed in resealable plastic bags (28 cm × 20 cm), and quickly brought back to the laboratory of Nanjing Forestry University (which took less than two hours from the collection site to the laboratory) to measure leaf fresh mass. **Table 1** provides the relevant data for the leaves collected for this study. **Figure 1** shows representative examples of the investigated leaves for the 12 species.

**Table 1:** Leaf collection information for the 12 species belonging to two families (Fagaceae and Ulmaceae) from Nanjing Forestry University campus, Nanjing, Jiangsu Province, P. R. China.

Species code	Family	Scientific name	Sampling date	Leaf type
1	Fagaceae	*Cyclobalanopsis glauca* (Thunberg) Oersted	26 August 2020	Evergreen
2	Fagaceae	*Lithocarpus glaber* (Thunb.) Nakai	25 August 2020	Evergreen
3	Fagaceae	*Quercus acutissima* Carr.	21 August 2020	Deciduous
4	Fagaceae	*Quercus aliena* Blume	20 August 2020	Deciduous
5	Fagaceae	*Quercus chenii* Nakai	27 August 2020	Deciduous
6	Fagaceae	*Quercus variabilis* Blume	20 August 2020	Deciduous
7	Ulmaceae	*Aphananthe aspera* (Thunb.) Planch.	2 September 2020	Deciduous
8	Ulmaceae	*Celtis julianae* Schneid.	31 August 2020	Deciduous
9	Ulmaceae	*Celtis sinensis* Pers.	1 September 2020	Deciduous
10	Ulmaceae	*Pteroceltis tatarinowii* Maxim.	3 September 2020	Deciduous
11	Ulmaceae	*Ulmus parvifolia* Jacq.	1 September 2020	Deciduous
12	Ulmaceae	*Zelkova serrata* (Thunb.) Makino	30 August 2020	Deciduous

### Leaf image processing

Leaf dry and fresh mass was measured using an electronic balance (ME204/02, Mettler Toledo Company, Greifensee, Switzerland; measurement accuracy 0.0001 g). Leaf dry mass was obtained after drying leaves in a ventilated oven (XMTD–8222; Jinghong Experimental Equipment Co., Ltd., Shanghai, China) at 80°C for at least 72 hours. To estimate leaf area, each fresh leaf was scanned with an Epson photo scanner (V550, Epson, Batam, Indonesia). Adobe Photoshop (version 9.0; Adobe, San Jose, CA, USA) was used to obtain black and white leaf edge images that were saved as bitmap images at a 600-dpi resolution. The protocols of Shi et al. (2018) and Su et al. (2019) were then used to calculate the pixel values of leaf images to obtain the planar coordinates of leaf boundary points. Leaf area was calculated by using the ‘bilat’ function in the ‘biogeom’ package (version 1.0.5; Shi et al., 2022b) based on R software (version 4.2.0; R Core Team, 2022).

### Statistical methods

A power-law function was used to describe the scaling relationships among DM, FM, and *A*:


(1) $$Y_{1}=\text{β}Y_{2}^{\alpha }$$

, where *Y*_1_ and *Y*_2_ represent interdependent variables (e.g., FM and *A*), β is the normalization constant, and α is the scaling exponent (Niklas, 1994). After log-log transformation, the power-law function was converted into


(2) y=γ+αx

, where *y* = ln(*Y*_1_), *x* = ln(*Y*_2_), and γ = ln (β). Parameters γ and α were estimated by using reduced major axis regression protocols (Niklas, 1994; Smith, 2009). The bootstrap percentile method (based on 3000 bootstrapping replicates) was used to test the significance of the difference in the estimated scaling exponents of *y* vs. *x* between any two of the 12 species (Efron and Tibshirani, 1993; Sandhu et al., 2011).

Ordinary least squares regression protocols were used to test the non-linearity of the log-transformed bivariate data. The linearity was rejected if the coefficient (γ_2_) of ^2^ of a quadratic model was statistically significant at the 0.05 significance level (see Zhao et al. (2019) for details).


(3) $$y={\text{γ}}_{0}+{\text{γ}}_{1}x+{\text{γ}}_{2}x^{2}$$

The percentage error (PE) was used to evaluate the effect of the non-linear term on the goodness of fit.


(4) $$\text{PE}=\frac{{\text{RSS}}_{linear\;}-{\text{RSS}}_{non-linear}}{{\text{RSS}}_{linear}}\times 100\%$$

, where RSS*_linear_* and RSS*_non-linear_* were the residual sum of squares of equations 2 and 3, respectively. As a rule of thumb, for two equations with similar model structures, if PE< 5%, the additional parameter (i.e., γ_2_) is not relevant.

The significance of differences in DM, FM, *A*, leaf absolute water content, leaf DM per unit area (LMA), and leaf FM per unit area (LFMA) among the 12 species were determined using the analysis of variance (ANOVA) based on the Tukey’s Honest Significant Difference (HSD) test at the 0.05 significance level (Hsu, 1996). All statistical analyses were performed using R (version 4.2.0) (R Core Team, 2022).

## Results

Statistically significant log-log DM vs. *A* and FM vs. *A* scaling relationships were observed for each of the 12 species (**Table 2**; **Figures S1, S2**). The numerical values of the scaling exponent of DM vs. *A* for 11 out of the 12 species exceeded unity and the lower bounds of the corresponding CIs of the scaling exponents for the 11 species exceeded unity. The only exception was *Aphananthe aspera* (Ulmaceae) whose goodness of fit (*r* ^2^ = 0.795) was lower than the majority of the other species. The numerical values of the scaling exponent of FM vs. *A* for all of the 12 species exceeded unity, and the corresponding 95% CIs of the scaling exponents did not include unity. For each species, the goodness of fit for the FM vs. *A* scaling relationship was higher than that for the DM vs. *A* scaling relationship, as reflected by the numerical values of the coefficients of determination (**Table 2**). For almost half of the species examined, the non-linear term was found to be significant. There were no significant differences between evergreen and deciduous species. The PE value, which designed whether it is worth introducing the non-linear term, was found to be smaller than 5% for each of most data sets (23/24). (**Table 2**; **Figures S1, S2**).

**Table 2:** Statistical parameters for dry mass vs. area and fresh mass vs. area in 12 species.

Species code	*n*	Diminishing returns	Equation	Slope CI	*r* ^2^	*P*_non-linearity_	PE (%)
1	364	Dry mass vs. area	*y* = –5.268 + 1.159 *x*	(1.115, 1.204)	0.861	<0.05	4.19
		Fresh mass vs. area	*y* = –4.313 + 1.111 *x*	(1.085, 1.137)	0.943	<0.05	1.53
2	357	Dry mass vs. area	*y* = –4.535 + 1.069 *x*	(1.028, 1.112)	0.855	0.8227	0.01
		Fresh mass vs. area	*y* = –3.857 + 1.065 *x*	(1.027, 1.106)	0.881	0.9787	0
3	346	Dry mass vs. area	*y* = –5.276 + 1.058 *x*	(1.020, 1.096)	0.867	0.3154	0.29
		Fresh mass vs. area	*y* = –4.837 + 1.132 *x*	(1.098, 1.166)	0.925	0.5759	0.09
4	346	Dry mass vs. area	*y* = –5.630 + 1.122 *x*	(1.096, 1.150)	0.956	<0.05	1.99
		Fresh mass vs. area	*y* = –4.612 + 1.101 *x*	(1.081,1.121)	0.980	<0.05	1.41
5	370	Dry mass vs. area	*y* = –5.544 + 1.122 *x*	(1.085,1.159)	0.890	0.7502	0.03
		Fresh mass vs. area	*y* = –4.858 + 1.134 *x*	(1.110, 1.158)	0.957	0.888	0.01
6	315	Dry mass vs. area	*y* = –5.740 + 1.201 *x*	(1.159, 1.247)	0.891	0.1119	0.81
		Fresh mass vs. area	*y* = –4.997 + 1.195 *x*	(1.160, 1.231)	0.935	0.0943	0.89
7	365	Dry mass vs. area	*y* = –5.454 + 0.997 *x*	(0.957, 1.039)	0.777	0.6695	0.05
		Fresh mass vs. area	*y* = –4.902 + 1.087 *x*	(1.046, 1.131)	0.854	<0.05	1.41
8	369	Dry mass vs. area	*y* = –5.673 + 1.159 *x*	(1.116, 1.208)	0.862	<0.05	2.60
		Fresh mass vs. area	*y* = –4.573 + 1.098 *x*	(1.071, 1.126)	0.950	<0.05	1.91
9	359	Dry mass vs. area	*y* = –5.139 + 1.116 *x*	(1.058, 1.176)	0.653	<0.05	1.68
		Fresh mass vs. area	*y* = –4.573 + 1.098 *x*	(1.071, 1.126)	0.758	<0.05	2.68
10	359	Dry mass vs. area	*y* = –5.930 + 1.174 *x*	(1.137, 1.213)	0.867	<0.05	2.01
		Fresh mass vs. area	*y* = –4.734 + 1.126 *x*	(1.100, 1.151)	0.940	<0.05	5.29
11	363	Dry mass vs. area	*y* = –6.176 + 1.520 *x*	(1.464, 1.584)	0.823	0.3377	0.26
		Fresh mass vs. area	*y* = –5.028 + 1.442 *x*	(1.399, 1.489)	0.914	0.2457	0.37
12	358	Dry mass vs. area	*y* = –5.495 + 1.168 *x*	(1.135, 1.202)	0.920	<0.05	2.44
		Fresh mass vs. area	*y* = –4.848 + 1.177 *x*	(1.153, 1.202)	0.959	<0.05	1.39

Species codes associated with binomials are provided in **Table 1**.

**Figure 2:**
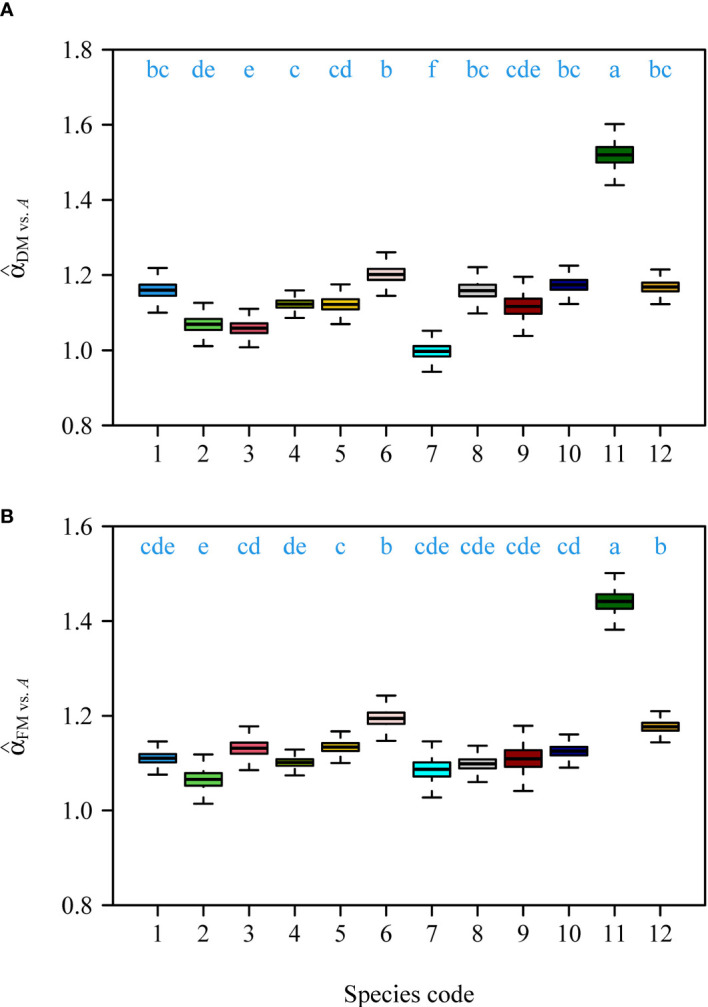
Comparisons of the estimated numerical values of the scaling exponents of DM vs. *A* among the 12 species **(A)**, and the estimated numerical values of the scaling exponents of FM vs. *A* among the 12 species **(B)**. The letters on the top of the whiskers signify the significance of the difference in the scaling exponent between any two pairs of the 12 species; the solid segments in the boxes represent the medians of the scaling exponents based on 3000 bootstrapping replications. Species codes associated with their binomials are the same as those in **Table 1**.

Interspecific comparisons among the two scaling exponents (i.e., for DM vs. *A*, and FM vs. *A*) showed that there were significant differences among the 12 species (**Figure 2**). Although there were slight differences between the interspecific scaling exponents of DM vs. *A* and those of FM vs. *A*, the variation trends were assessed to be the same for the 12 species (**Figure 2**).

**Figure 3:**
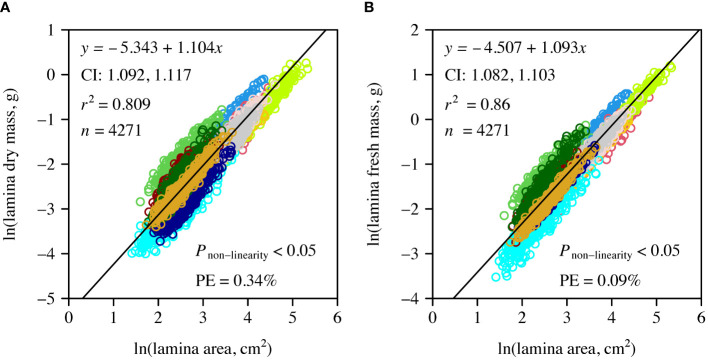
Log-log bivariate plots and linear fits to leaf dry mass vs. *A* **(A)**, and to leaf fresh mass vs. *A* **(B)** for the pooled data of the 12 species. Open circles are the observed data; the straight lines are the log-log regression curves.

The numerical values of the scaling exponents of DM vs. *A* and of FM vs. *A* and the corresponding CIs for the pooled data were greater than unity. However, the numerical value of the latter was slightly smaller than that of the former (**Figure 3**). In addition, the coefficient of determination of DM vs. *A* was smaller than that of FM vs. *A*.

The non-linear terms of the allometric model were found to be significant for both DM vs. *A* and FM vs. *A*. However, the PE values were < 5%, which indicated that the non-linear term did not improve the model fit (**Figure 3**).

**Figure 4:**
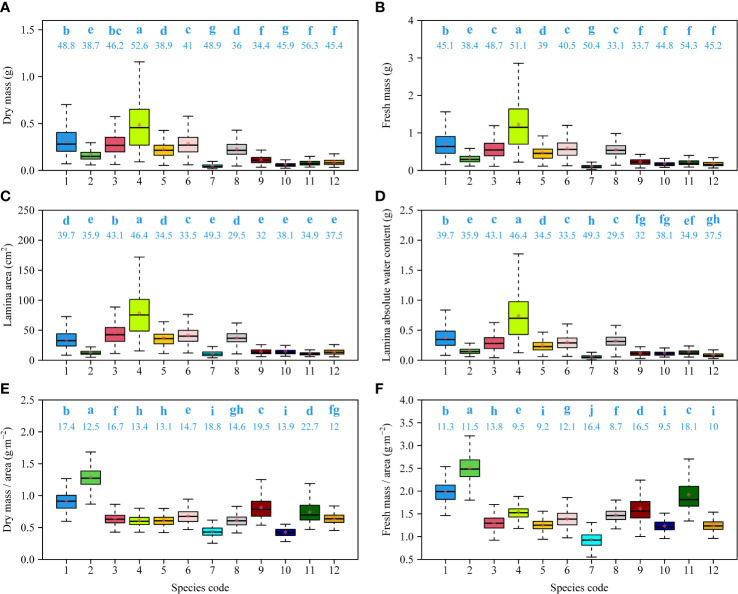
Comparisons of dry mass **(A)**, fresh mass **(B)**, lamina area **(C)**, lamina absolute water content **(D)**, ratio of fresh mass to area **(E)**, and ratio of dry mass and area **(F)** among 12 species. The letters on the top of the whiskers of the boxes signify the significance of the difference between any two of 12 species; the numbers on the top of the whiskers of the boxes signify the coefficients of variation of leaf measures; the solid segments and asterisks within the boxes represent the medians and means of each leaf measure based on 3000 bootstrapping replications, respectively. Species codes associated with their binomials are the same as those in **Table 1**.

There were significant differences in leaf size (as measured by DM, FM, or *A*) among the 12 species. The numerical values of Fagaceae species were generally greater than those of Ulmaceae species (**Figures 4A–C** **)**. Leaf absolute water content had a similar trend with respect to leaf size, and the leaves of the Fagaceae species tended to have a higher water content (**Figure 4D**).

There were significant differences in leaf DM per unit area (LMA) and leaf FM per unit area (LFMA) among the 12 species. Two evergreen species, *Cyclobalanopsis glauca* and *Lithocarpus glaber*, had the largest LMA and LFMA values (**Figures 4E, F** **)**.

## Discussion

The leaves of evergreen and deciduous species both conformed to the phenomenon called diminishing returns as indicated by the numerical values of the exponents of leaf mass (DM or FM) vs. *A* exceeding unity. The exponent of FM vs. *A* was numerically slightly lower than that of DM vs. *A*, likely because the addition of water to the mass of the leaf lamina. An examination or the non-linear response in diminishing returns indicated that the non-linear model was not superior and was less parsimonious compared to the linear model as indicated by higher *r* ^2^ and PE values.

No significant difference in DM, FM, and *A* was observed between the ten deciduous and the two evergreen species. However, leaf dry mass per unit area (LMA) and leaf fresh mass per unit area (LFMA) differed between the evergreen and deciduous species. Specifically, the evergreen species had larger mass investments in their per unit leaf area than the other deciduous species.

We conclude therefore that FM vs. *A* is a more reliable than DM vs. *A* for describing leaf scaling relationships of *M* vs. *A* with respect to both the evergreen and deciduous species. The leaf biomass investment strategy between evergreen and deciduous species is slightly different, but they both conformed to diminishing returns. However, it worth noting that the concept of diminishing returns revolves around the investments made in the construction of leaves and not around the mechanical loads that a leaf must support (Niklas et al., 2007). The superiority of the reliability of FM vs. *A* highlights the importance of mechanical support, whereas diminishing returns in the context of DM vs. *A* highlights the importance of carbon allocation (Niklas and Spatz, 2012). With this distinction in mind, we address in the following sections each of the two questions outlined in the Introduction.

### Is leaf FM vs. *A* more reliable than DM vs. *A* when assessing diminishing returns

The two evergreen species manifested a FM vs. *A* scaling relationship that was statistically more robust than that of the DM vs. *A* scaling relationship (see also Huang et al., 2019a; Huang et al., 2019b; Liu et al., 2020). Among each of the ten deciduous species and the two evergreen species, the goodness of fit (as gauged by *r* ^2^) for the FM vs. *A* scaling relationship was greater than that of DM vs. *A* scaling relationship (**Table 2**), as reported for other evergreen species (Huang et al., 2019a; Huang et al., 2019b; Liu et al., 2020; Guo et al., 2021).

In prior studies, reduced major axis protocols have been used to assess the log-log bivariate relationships of biologically interrelated variables of interest. However, to date, there are no available methods to test the statistical significance of the non-linear term based on reduced major axis protocols. In the present study, ordinary least squares regression protocols were used to test the significance of the non-linear term (see also Zhao et al., 2019). Although the non-linear terms were found to be significant for the *M* vs. *A* scaling relationship for some datasets, the PE values nevertheless showed that the addition of the non-linear term did not improve the significance of correlations and unnecessarily increased the model complexity and was, therefore, unnecessary and a distraction

It is obvious that relationship between FM and lamina *A* depends on both the leaf water content and the dry mass content across all of the species examined in this study. FM depends on the availability of water in the local environment, whereas leaf dry mass is unresponsive to variations in water availability after leaf expansion (Jiao et al., 2022). For this reason, all of the leaves used in the present study were sampled at the same season and daytime of day in an effort to limit the effect of rainfall on leaf fresh mass and its effect on the *r* ^2^ of FM vs. *A*. Because leaf area determines the ability to capture light to a great extent, the leaf biomass invested in the construction of lamina area and thickness is considered to be a trade-off between the ability to capture and utilize light (White and Montes-R, 2005; Jullien et al., 2009; Koester et al., 2014). Thicker leaves tend to have a higher water content, longer palisade cells, and multiple palisade cell layers, which can enhance area-based photosynthesis (Mitchell et al., 1999; Lambers et al., 2008).

Therefore, we conclude that FM vs. *A* is a better measure of leaf performance than DM vs. *A* when considering the scaling of leaf biomass with respect to *A* (Liu et al., 2020).

### Does the leaf biomass investment strategy differ between deciduous and evergreen species?

Leaf dry mass, fresh mass, and water content does not differ significantly between the evergreen and deciduous species examined in this study (**Figures 4A–C**). This indifference might reflect equivalent investments to maintain essential metabolic pathways regardless of leaf life span (Niinemets et al., 2006; Yan et al., 2016). Regardless of the proximate cause(s), we cannot conclusively confirm whether deciduous species invest more or less biomass to leaf area expansion compared to evergreen species.

However, our data indicate that the LMA and FLMA of deciduous species are significantly smaller than those of evergreen species (**Figures 4E, F**; see also Poorter et al., 2009). LMA is frequently used as a surrogate measure of photosynthetic rate and growth strategy (Wright et al., 2004; Poorter et al., 2009). Typically, photosynthetic activity per leaf area declines with increasing LMA, whereas the concentration of proteins and minerals tends to increase as LMA decreases. This trend is attended by lower concentrations of lignin and other secondary compounds, which leads to increased carbon return rates but also to lower leaf life-span (Lambers and Poorter, 1992; Wright and Westoby, 2002). Compared to deciduous species, evergreen species generally have higher LMA and possess a larger proportion of non-photosynthetically active tissue associated with longer leaf life spans. The evergreen species included in our study occur in shaded environments with low soil moisture that demand long-lasting and thick leaves with high LMA. In contrast, the deciduous species occuy habitats with higher light and water availabilities permitting faster life history strategies expressed by, among other things, low LMA values (see also Poorter et al., 2009). Based on these results, we conclude that deciduous species may invest more biomass in their photosynthetically active tissues per unit leaf area to balance the shorter duration of their investment returns. Noting that evergreen species possess larger LMA and LFMA than deciduous species, we also speculate that these species invest more biomass in the construction of non-photosynthetically active tissues to support their longer duration of investment returns.

## Conclusions

The data presented here indicate that FM vs. *A* is more reliable for describing leaf scaling relationships than DM vs. *A* for both evergreen and deciduous species. The FM vs. *A* scaling exponents of the 12 species investigated have 95% CIs numerically greater than unity and a similar trend is observed for DM vs. *A*. Thus, the results are consistent with the phenomenon called “diminishing returns”. The data also indicate that the leaves of evergreen species have higher LMA and LFMA values than the deciduous species within the same family (Fagaceae). These results indicate that the leaf biomass investment strategies of deciduous and evergreen species are slightly different: deciduous species tend to obtain larger light harvesting capabilities by investing less biomass per unit area. Future research is required to determine if these trends apply to species within other families, particularly families including evergreen and deciduous species.

## Data availability statement

The datasets presented in this study can be found in online repositories. The names of the repository/repositories and accession number(s) can be found below: Dryad Data Repository, https://doi.org/10.5061/dryad.kprr4xh4r.

## Author contributions

KN, PS, and JS designed this work; YL carried out the experiment; PS analyzed the data; XG and KN wrote the initial draft, and contributed equally to this work; JS, PS and JX commented on and revised the manuscript; all authors read and agree with this manuscript.

## Funding

JX was supported by Jiangsu Forestry Science & Technology Innovation and Extension Project (No: LYKJ[2022]02); JS acknowledges funding by the German Research Foundation (DFG) with a research scholarship (No: SCHR1672/1- 1) and by a Macquarie University Research Fellowship.

## Acknowledgments

We thank Yabing Jiao, Ülo Niinemets and Kexin Yu for their valuable help in the preparation of this manuscript. We also thank Drs. Boris Rewald and Tiina Tosens for handling our manuscript and two reviewers for providing valuable comments on the earlier version of this manuscript.

## Conflict of interest

The authors declare that the research was conducted in the absence of any commercial or financial relationships that could be construed as a potential conflict of interest.

## Publisher’s note

All claims expressed in this article are solely those of the authors and do not necessarily represent those of their affiliated organizations, or those of the publisher, the editors and the reviewers. Any product that may be evaluated in this article, or claim that may be made by its manufacturer, is not guaranteed or endorsed by the publisher.
