# Supplementary material for: Diminishing returns: A comparison between fresh mass vs. area and dry mass vs. area in deciduous species
Source: Front Plant Sci. 2022 Oct 4;13:832300. doi: 10.3389/fpls.2022.832300 (PMC9576923; doi:10.3389/fpls.2022.832300)
Supplement: Supplementary file 1 [file DataSheet_1.docx]

**Supplementary Information**

**“Diminishing returns”: a comparison between fresh mass vs. area and dry mass vs. area in deciduous species**

**Xuchen Guo^1^, Karl J. Niklas^2^, Yirong Li^3^, Jianhui Xue^1,4^*, Peijian Shi^1,^*, and Julian Schrader^5,6,^***

^1^ Bamboo Research Institution, College of Biology and the Environment, Nanjing Forestry University, Nanjing 210037, China

^2^ School of Integrative Plant Science, Cornell University, Ithaca, NY 14853, USA

^3^ College of Life Science, South China Agricultural University, Guangzhou, China,

‎^4^ Institute of Botany, Jiangsu Province and Chinese Academy Sciences, Nanjing 210014, China

^5^ School of Natural Sciences, Macquarie University, Sydney, NSW, Australia

^6^ Biodiversity, Macroecology and Biogeography, University of Göttingen, Göttingen 37077, Germany

*** Correspondence:**

Jianhui Xue, jhxue@njfu.edu.cn

Peijian Shi, pjshi@njfu.edu.cn

Julian Schrader, jschrader@posteo.de


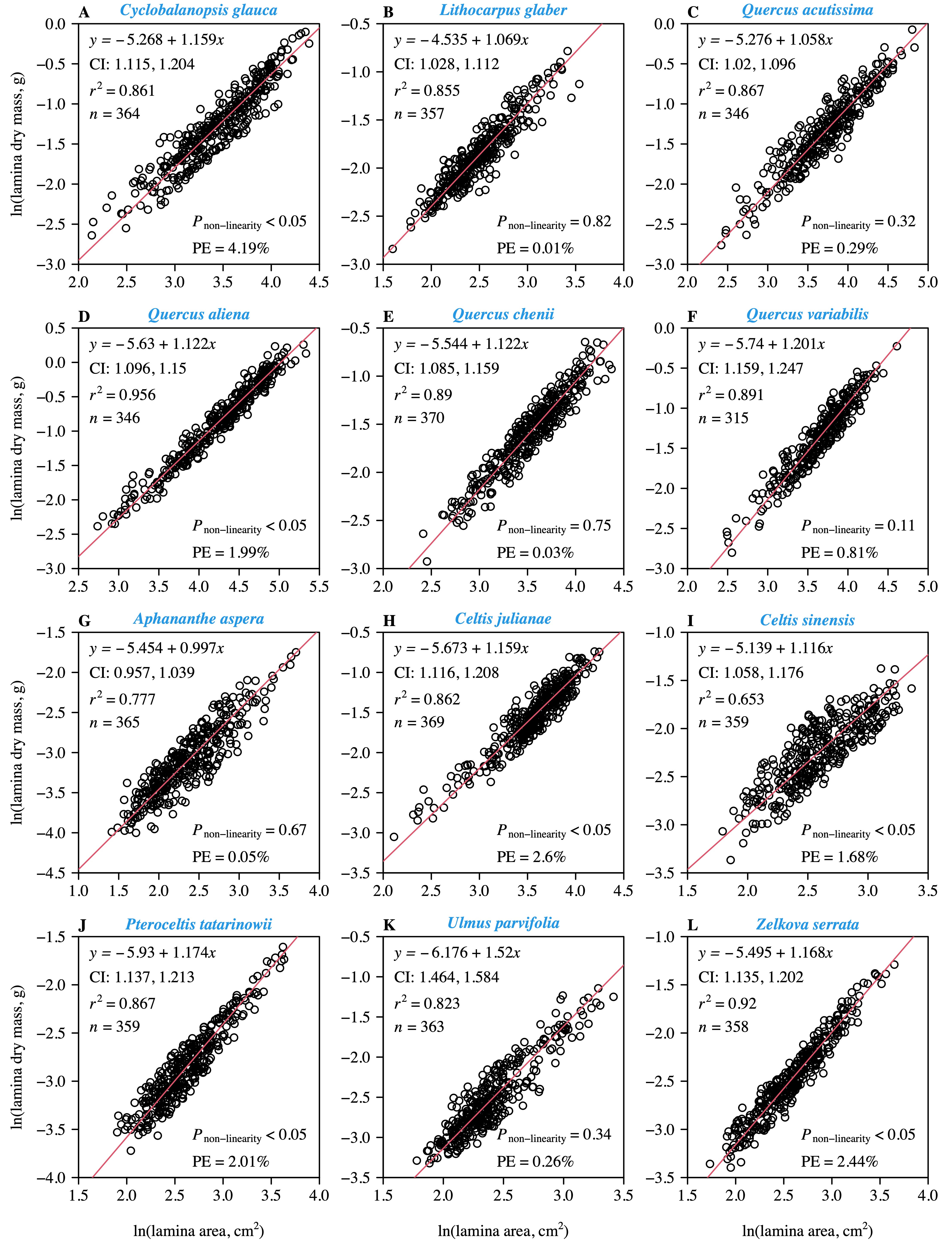


**FIGURE S1** Log-log bivariate plots of leaf dry mass vs. leaf area for each of the 12 species. Open circles are observed values; red lines are log-log regression curves.


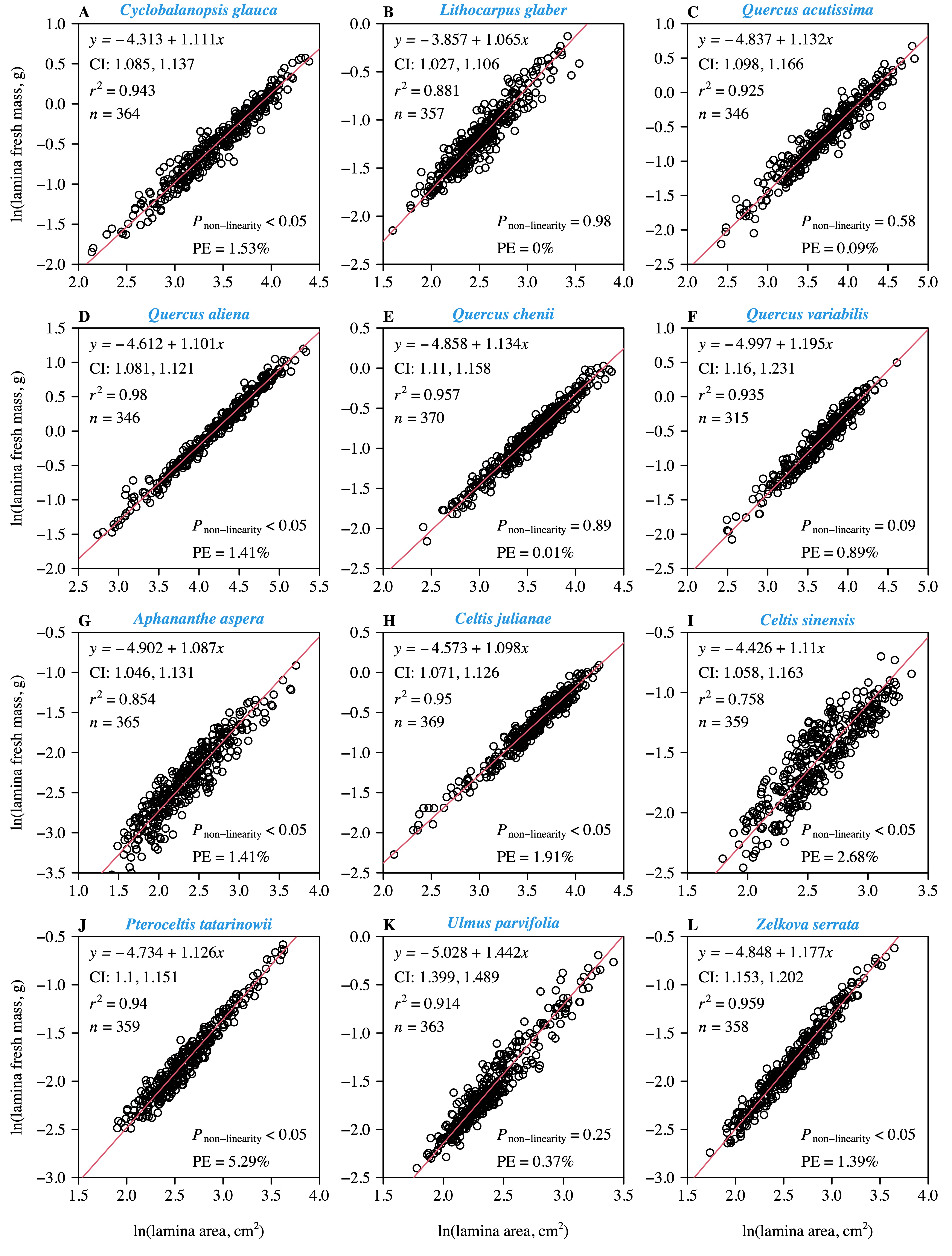


**FIGURE S2** Log-log bivariate plots of leaf fresh mass vs. leaf area for each of the 12 species. Open circles are observed values; red lines are log-log regression curves.
